# Digital mental health: Perceptions and opinions of Tunisian patients

**DOI:** 10.1192/j.eurpsy.2023.398

**Published:** 2023-07-19

**Authors:** H. Jemli, U. Ouali, M. Hajri, R. Jenhani, M. Djelassi, R. Jomli

**Affiliations:** ^1^Psychiatry deparment A, Razi Hospital, Manouba; ^2^Faculty of medicine of Tunis, University Tunis Elmanar, Tunis; ^3^Outpatient psychiatric department, Razi Hospital, Manouba, Tunisia

## Abstract

**Introduction:**

With the rapid advancement of modern technology, many countries have adopted mental health care systems supplemented by digital means of communication. Are Tunisian patients “ready” for the “digital revolution”?

**Objectives:**

The aim of our study was to assess perceptions of people living with mental illness on digital mental health.

**Methods:**

We developed a cross-sectional study where we randomly included patients who were treated for a psychiatric disorder in a public or a private practice. Inclusion criteria were: subject 18 years old or older, clinical remission for at least three months. We developed a questionnaire on sociodemographic and clinical variables. We also included questions on patients’ level of interest in using digital mental health services such as teleconsultation and mental health smartphone apps. Perceived obstacles in using digital mental health by patients were also evaluated.

**Results:**

Our sample size was 260 patients. The mean age of our population was 36,4 years old with. The mean distance from the household to the mental health care provider was 17,3 km.

Two thirds of the sample had access to a wifi connection at home (172 patients). When asked about the content of internet searches, 66% have already looked for information on their mental health or mental disorders on web pages. Patients were very interested in video teleconsultation with their therapists (72%), psychoeducation apps (68%) and online mood journals (61%). They expressed little to no interest in online exchanges with other patients and medication reminder apps. The most reported obstacles in implementing digital mental health as noted by patients were : lack of perceived effectiveness, virtual communication with their therapist and confidentiality issues.

**Conclusions:**

Mental health patients in Tunisia expressed a great interest in teleconsultations and online psychoeducation programs. Further research on the willingness of mental health professionals to adopt digital mental health services are needed.

**Disclosure of Interest:**

None Declared

